# High Glucose Level Disturbs the Resveratrol-Evoked Curtailment of CX3CL1/CX3CR1 Signaling in Human Placental Circulation

**DOI:** 10.1155/2017/9853108

**Published:** 2017-06-01

**Authors:** Dariusz Szukiewicz, Michal Pyzlak, Grzegorz Szewczyk, Aleksandra Stangret, Seweryn Trojanowski, Michal Bachanek, Wojciech Braksator, Jaroslaw Wejman

**Affiliations:** ^1^Department of General & Experimental Pathology with Centre for Preclinical Research and Technology (CEPT), Second Faculty of Medicine, Medical University of Warsaw, ul. Pawinskiego 3C, 02-106 Warsaw, Poland; ^2^Department of Obstetrics & Gynecology, Second Faculty of Medicine, Medical University of Warsaw, ul. Kondratowicza 8, 03-242 Warsaw, Poland; ^3^Department of Cardiology, Hypertension, and Internal Medicine, Second Faculty of Medicine, Medical University of Warsaw, ul. Kondratowicza 8, 03-242 Warsaw, Poland; ^4^Department of Pathology, Professor Witold Orlowski Public Clinical Hospital, Medical Center for Postgraduate Education, ul. Czerniakowska 231, 00-416 Warsaw, Poland

## Abstract

Hyperglycemia-induced hyperactivity of chemokine CX3CL1 (fractalkine) occurs in the human placenta. Anti-inflammatory/antioxidant activities of resveratrol (3,5,4′-trihydroxy-*trans*-stilbene) are related to the modulation of chemokine CX3CL1 and its receptor, CX3CR1, signaling pathways. We examined the influence of high glucose (25 mmol/L glucose; HG group; *N* = 36) on resveratrol-mediated effects on CX3CL1 and TNF-*α* production by the placental lobule, CX3CR1 expression and contents of CX3CR1, TNF-*α* receptor 1 (TNFR1), and NF-*κ*B proteins in placental tissue. The placental lobules perfused under normoglycemic conditions formed the control NG group (*N* = 36). Resveratrol (50 and 100 *μ*M; subgroups B and C) administered into the perfusion fluid lowered the production of both CX3CL1 and TNF-*α*. The reductions in CX3CL1 levels were more evident in the NG group. CX3CR1 expression was significantly higher in the NG subgroups B and C compared to the HG subgroups B and C (385.2 and 426.5% versus 199.3 and 282.4%, resp.). An increase in CX3CR1 protein content in placental lysates was observed in the NG subgroups B and C. Also, resveratrol significantly decreased NF-*κ*Bp65 protein content only in the NG group, not affecting hyperglycemia-elicited TNFR1 upregulation. In conclusion, euglycemia assures optimal effects of resveratrol pertaining to CX3CL1/CX3CR1 signaling in the placenta. Future studies on resveratrol are needed, especially those including maternal-fetal risk assessments.

## 1. Introduction

Optimal control of glycemia should be considered in the context of homeostasis, including the preservation of a stable cytokine network in the vascular system [1]. A change in the balance between proinflammatory and anti-inflammatory cytokines, with increased release of the chemokine (chemotactic cytokine) CX3CL1 (fractalkine and neurotactin), in response to high-glucose conditions has been observed in many tissues and organs [2, 3]. Such an inflammatory background may play a role in the pathogenesis and pathophysiology of diabetes [2, 4]. Abnormal CX3CL1 signaling is associated with increased angiogenesis and accelerated atherosclerosis in hyperglycemic conditions [5, 6]. Moreover, CX3CL1 is considered a potential marker of the deterioration of pancreatic *β*-cell function in the islets of Langerhans [7]. Serum levels of CX3CL1 are associated with markers of insulin resistance in gestational diabetes [8]. Interestingly, it was recently demonstrated in mice that the deficiency of the CX3CL1 receptor CX3CR1 exerts a protective effect on glucose intolerance and insulin resistance [9]. The persistence of high glucose levels in diabetes and prediabetes promotes the production of reactive oxygen species, which aggravate oxidative stress and endothelial dysfunction [10].

Changes in the cytokine profile in response to hyperglycemia have been reported in placental circulation in diabetic pregnancies [11]. Since human placental vessels lack autonomic innervation, it is important to note that the humoral effects of vasoactive compounds (e.g., nitric oxide) that are produced locally by placental cells dominate the regulation of feto-placental blood flow, thus influencing vascular resistance [12]. Despite some controversies, both clinical observations and histopathological studies of the human placenta have confirmed an increased incidence of inflammatory reactions in diabetes [5, 11, 13]. Considering that the most important functions of the human placenta include immunosuppression to protect the fetal “allograft” from T-cell-mediated immune aggression and infectious agents, the placental chemokine CX3CL1 may deserve special attention [14]. Together with certain other chemokines (CCL4, CCL7, and CCL14), CX3CL1 is involved in the processes of implantation, trophoblast invasion into the spiral uterine arteries, placental angiogenesis, response to inflammatory and immunologic factors within the utero-placental unit, and induction of labor [15–17].

Chemokine (C-X3-C motif) ligand 1 (CX3CL1) is encoded on the long arm of human chromosome 16 at position 13 and contains three exons. Unlike other chemokines, CX3CL1 is of nonhematopoietic origin; currently, CX3CL1 is the lone member of the CX3C subfamily of chemokines (*δ*-chemokines) and possesses three amino acid residues between the first two cysteine residues [18]. CX3CL1 is synthesized as a membrane molecule consisting of the 76-amino acid N-terminal chemokine domain, a 241-amino acid-glycosylated mucin-like stalk, a 21-amino acid hydrophobic transmembrane region, and a 37-amino acid intracellular C-terminal domain [19]. This protein can be cleaved by the lysosomal cysteine protease, cathepsin S, and by the members of the disintegrin and metalloproteinase (ADAMs) family, such as ADAM-10 and ADAM-17, which release a soluble form of CX3CL1 that contains the chemokine domain and the glycosylated mucin-like stalk [20]. Thus, CX3CL1 exists in a soluble form as a chemotactic protein for monocytes, natural killer (NK) cells, and T cells (but not neutrophils) and in a membrane-anchored form, mainly on endothelial cells, as a cell adhesion molecule. This set of properties, both a chemoattractant and an adhesive compound, makes CX3CL1 unique among other chemokines [21]. Moreover, CX3CL1 exerts cytotoxic effects on endothelial cells [22], which express substantial levels of CX3CL1; considering the additional synergistic effects of TNF-*α* and IFN-*γ*, almost every stimulus that influences cell homeostasis may induce CX3CL1 secretion [21, 22]. The biological activities of CX3CL1 are mediated by its sole receptor CX3CR1 (previously denoted as V28), a G*α*i protein-linked seven-transmembrane receptor [19]. This receptor is localized on the surface of endothelial cells, mast cells, monocytes, macrophages, NK cells, platelets, subpopulations of T-lymphocytes, microglial cells, neurons, and smooth muscle cells [23]. In humans, for example, low surface expression of CX3CR1 is an indicative of the CD14^++^ and CD16^−^ monocyte subset, whereas high CX3CR1 expression corresponds to CD14^+^ and CD16^+^ monocyte populations [23, 24].

The stimulation of CX3CR1 will activate CX3CL1-dependent and integrin-dependent migrations of cells with augmented adhesion as a result of synergistic reactions [23, 24]. Autoregulatory interactions between CX3CL1 and CX3CR1 may be driven by local changes in CX3CR1 expression. It was suggested that CX3CL1 autoactivates its own expression via the PI3-kinase/PDK1/Akt/NIK/IKK/nuclear factor kappa B (NF-*κ*B) signaling pathway [25]. Tumor necrosis factor alpha (TNF-*α*) also induces the expression of CX3CL1 and CX3CR1 in rat aortic smooth muscle cells via the NF-*κ*B pathway [26]. The human protein encoded by the TNFRSF1A gene is one of the major receptors for TNF-*α*, and TNFR1 (CD120a) can be detected in almost all cell types, including endothelial cells [26, 27]. Signaling induced by the stimulation of TNFR1 is involved in the activation of NF-*κ*B transcription, the modulation of inflammatory responses, and apoptosis [27].

Resveratrol, a trihydroxy derivative of stilbene (3,5,4′-trihydroxy-*trans*-stilbene) that exists in isomeric *trans* and *cis* forms, is a naturally occurring polyphenolic phytoalexin that can be isolated from red grapes (predominantly from grape skin), berries, peanuts, and red wine [28]. This compound possesses anti-inflammatory and antioxidant properties related to its interference with the NF-*κ*B and JAK/STAT signaling pathways, which regulate the expression of various genes involved in inflammation, including CX3CL1 [28, 29]. Moreover, it has been demonstrated that resveratrol suppresses TNF-*α*-induced CX3CL1 activity in endothelial cells [30]. The abovementioned properties may be of particular therapeutic relevance in metabolic disorders with an inflammatory background. The harmful effects of hyperglycemia-related changes in the cytokine profile have been observed in placental circulation, including in endothelial cells and vascular smooth muscle cells [11, 31].

In this study, we examined the influence of resveratrol on CX3CL1 and TNF-*α* production in extracorporeally perfused human term placental lobules under hyperglycemic (25 mmol/L glucose; HG group; *N* = 36) and normoglycemic (5 mmol/L glucose; NG group; *N* = 36) conditions. Endothelial CX3CR1 expression in the placental microvasculature and contents of CX3CR1, TNFR1, and NF-*κ*B proteins in the perfused placental tissue were also examined in the studied groups.

## 2. Materials and Methods

The study was conducted in compliance with the international and local laws of human experimentation, and the project was officially approved by the local Ethics Committee of the Medical University of Warsaw (Ethical Clearance Approval no. KB21/2011). A written informed consent has been obtained from each woman whose placenta was used in the study. Strictly speaking, this work has been carried out in accordance with the Code of Ethics of the World Medical Association (Declaration of Helsinki) for experiments involving humans, and the Uniform Requirements for manuscripts submitted to Biomedical Journals have been fulfilled.

### 2.1. Placental Collection

The thirty-six heparinized placentae included in this study were obtained from previously nulliparous women after normal-course, singleton pregnancies delivered by elective cesarean section at term. The indications for cesarean section included breech presentation of the fetus and high-grade myopia in pregnant woman. The placentae were subjected to dual in vitro perfusion at 37°C using a modified Schneider's method [32, 33]. Two nonadjacent lobules of similar size were selected and isolated from each placenta for simultaneous perfusion; the first lobule was exposed to hyperglycemic perfusion fluid (HG group), and the other lobule was subjected to perfusion with a normal concentration of glucose (NG group). More detailed clinical characteristics of these two homogenous groups are provided in Table 1. The chorionic vessels in the two selected lobules were cannulated immediately following delivery, and the placenta was transported on ice to the laboratory in a plastic box filled with cold, sterile phosphate-buffered saline (PBS). The perfusion was started not more than 20 min from the time of placental detachment.

### 2.2. In Vitro Perfusion of the Placental Lobules, Administration of Resveratrol

**Figure 1:**
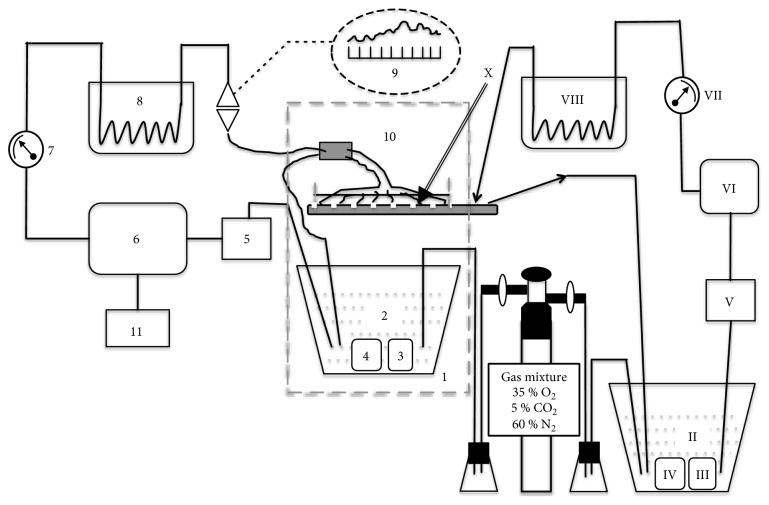
General scheme of in vitro dual placental perfusion system under normoxia. Elements of fetal side (Arabic numeration) and maternal side (Roman numeration) perfusion system: 1, I thermostatic chamber; 2, II reservoir; 3, III oxygen monitor with polarographic (Clark) oxygen electrodes; 4, IV pH meter; 5, V filter; 6, VI peristaltic pump; 7, VII flowmeter; 8, VIII thermostat; 9 Ludwig's manometer with kymograph; 10, X fetal and maternal compartments of perfusion chamber, 11 dosimetric pump.

Each isolated placental lobule (two per placenta) was bilaterally perfused. A diagram of the two-sided closed extracorporeal perfusion system is shown in Figure 1. The experimental technique used in this study has been described in detail elsewhere [33]. Briefly, isotonic perfusion fluid (Krebs-Ringer with 200 mg/L cefazolin and 2 mg/L gentamicin) was buffered at pH 7.4 (in PBS), and the extracorporeal fetal-side circuit perfusion process was strictly monitored to maintain perfusion pressure, flow stability, perfusion fluid volume, and hydrogen ion concentration within the range of allowable fluctuations, as shown in Table 2.

The perfusion fluid was enriched with a gas mixture containing 35% O_2_, 5% CO_2_, and 60% N_2_. The oxygen concentration applied in the gas mixture provided a partial pressure of oxygen (*p*O_2_) in the perfusion fluid of 13.3 kPa. The saturation value of dissolved oxygen was monitored throughout the experimental period by sampling the perfusion fluid at 30 min intervals between 30 and 120 min of perfusion. Polarographic Clark-type oxygen electrodes were mounted in the flow-through thermostatic chambers on both the fetal and maternal sides of the perfusion system (Figure 1). This type of *p*O_2_ electrode consists of an anode and cathode and measures oxygen tension amperometrically; the *p*O_2_ electrode produces a current at a fixed polarizing voltage (−700 mV), which is directly proportional to the partial pressure of oxygen (*p*O_2_) in the perfusate. The current evoked is a result of the subsequent reduction of oxygen at the cathode. The oxygen probes [Yellow Springs Instruments (YSI), IL, USA, model 05520-16] delivered the current to the two-channel biological oxygen monitor (YSI, model 5300A), which was connected to a computer-based data acquisition system.

*Trans*-tris methoxy resveratrol was purchased from Cayman Chemical Comp., USA, (catalogue number 10188) as a crystalline solid. For maximum solubility in aqueous buffers, *trans*-tris methoxy resveratrol was dissolved in dimethyl sulfoxide (DMSO) and then diluted with PBS to obtain the three desired concentrations (10, 50, and 100 *μ*M) for the respective subgroups (A, B, and C). After 60 min of placental perfusion, 5 mL boluses of resveratrol was administered into the circuits on both the fetal and maternal sides at 30 min intervals.

The respective hyperglycemic and normoglycemic controls perfused without resveratrol have been established during the preliminary phase of this study.

### 2.3. Measurements of CX3CL1 and TNF-*α*

During the 150 min perfusion, including the initial 30 min adaptive phase, basal [immediately before the administration of lipopolysaccharide (LPS)] and LPS-evoked (10 ng/mL) CX3CL1 released into the fetal-side placental circulation was examined quantitatively in perfusion fluid samples by ELISA. The RayBio^®^ Human Fractalkine ELISA Kit (RayBiotech Inc., USA, catalogue number ELH-Fractalkine) applied in this study has a very high specificity that exceeds most other available ELISA tests for the detection of CX3CL1 in placental perfusates, to the best of our knowledge. According to the manufacturer's information, the minimum detectable dose of CX3CL1 for this test is typically less than 300 pg/mL, and cross-reactivity was not observed with any of the cytokines tested including human angiogenin, BDNF, BLC, ENA-78, FGF-4, IL-1*α*, IL-1*β*, IL-2, IL-3, IL-4, IL-5, IL-7, IL-8, IL-9, IL-10, IL-11, IL-12 p70, IL-12 p40, IL-13, IL-15, IL-309, IP-10, G-CSF, GM-CSF, IFN-*γ*, leptin, MCP-1, MCP-2, MCP-3, MDC, MIP-1*α*, MIP-1*β*, MIP-1*δ*, PARC, PDGF, RANTES, SCF, TARC, TGF-*β*, TIMP-1, TIMP-2, TNF-*α*, TNF-*β*, TPO, and VEGF (see Abbreviations). The samples of the perfusion fluid were collected every 30 min from 30 to 150 min. The mean values for each group and at each time point were calculated.

The dose of LPS administered in this study was precisely titrated during the preliminary phase of the perfusion experiments. Based on the series of dose-response approaches (the dose-response curve analyses), 10 ng/mL was established as the mean dose of LPS eliciting 45–55% maximal TNF-*α* response (data not shown). Thus, the administration of LPS was an approach to standardize the inflammatory response.

In addition to CX3CL1 assessments, TNF-*α* levels in the perfusate samples obtained at the same time points were examined. Commercially available kit was used (RayBio Human TNF-alpha ELISA Kit; catalogue number ELH-TNFalpha-001) according to the manufacturer's instructions. The minimum detectable dose of TNF-*α* amounted to less than 10 pg/mL.

As a form of control in relation to these above measurements (CX3CL1 and TNF-*α*), we used our unpublished data from the previous perfusions of 16 placental lobules (8 with hyperglycemic and 8 with normoglycemic perfusion fluid) in the same manner, except the administration of resveratrol.

### 2.4. Detection and Quantification of NF-*κ*B in Perfused Placental Tissue

To compare the influence of different doses of resveratrol on NF-*κ*B signaling in the high-glucose and normal-glucose groups, NF-*κ*Bp65 protein content was quantified in placental (perfused lobule) tissue homogenates following the termination of the perfusion experiments. The NF-*κ*Bp65 (total) ELISA kit (Invitrogen Corp., Camarillo, CA, USA; catalogue number KHO0371) designed to detect and quantify the level of NF-*κ*Bp65 regardless of phosphorylation state was used. Before obtaining a lysate, the tissue was cut into approximately 1 mm^3^ cubes using a razor blade on a glass plate on ice. To gently disrupt the cells, the cubes were transferred into a handheld Potter S homogenizer (Sartorius™ Stedim Biotech GmbH). Ice-cold phosphoprotein lysis buffer containing 4 mM sodium pyrophosphate, 50 mM HEPES, 100 mM NaCl, 10 mM EDTA, 10 mM NaF, 2 mM NaVO_4_, 1 mM PMSF, 10% Triton X-100, 5 *μ*g/mL leupeptin, and 5 *μ*g/mL aprotinin was used. The liquefied tissue was poured into 1.5 mL tubes, and the tissue and cell debris were removed by centrifugation at 10,000 × g for 3 min at 4°C. The cleared supernatant was subjected to in vitro quantitative measurements of NF-*κ*Bp65 protein using 96-well plates and a microplate reader. Based on the information provided by the manufacturer, the NF-*κ*Bp65 protein levels detected in this manner are consistent with the results obtained by Western blot analysis. The p50 (NF-*κ*B1)/p65 (RelA) heterodimer assessed in this ELISA kit is the most abundant form of NF-*κ*B [34]. The assay has no cross-reactivity with either NF-*κ*Bp50 or I*κ*B*α*, but cross-reactivity with other NF-*κ*B family members was not evaluated. The minimum detectable level of NF-*κ*Bp65 protein was 50 pg/mL, and the detection range was 78–5000 pg/mL.

**Figure 2:**
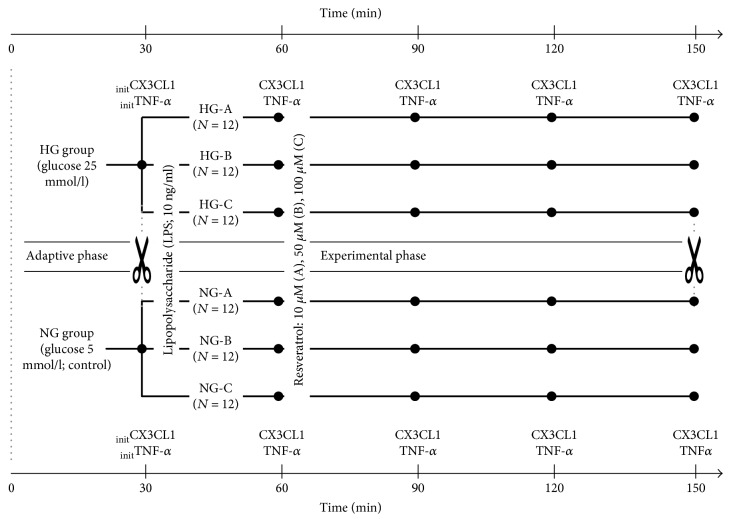
The experimental setup of the perfusion procedures with measurement time points for CX3CL1 and TNF-*α* (marked with dots). The initial concentrations of CX3CL1 and TNF-*α* were measured at the end of adaptive phase (_init_CX3CL1 and _init_TNF-*α*, resp.). Time points for the collection of the placental specimens are marked with scissors icon.

The experimental setup for the perfusion procedures, the administration of LPS and resveratrol, and the CX3CL1 and TNF-*α* measurements at fixed time points is presented in Figure 2.

### 2.5. CX3CR1 Immunostaining

After completing the perfusion procedure, a single specimen was excised in a standardized manner from each perfused lobule from the region contiguous with the fetal surface of the placenta. Following formalin fixation and paraffin embedding, three 5 *μ*m sections were obtained from each specimen/examined lobule. The methodology for the in vitro experiment precluded obtaining placental tissue specimens during the perfusion phase. Thus, the data on CX3CR1 expression were not collected at the same time points as those for the CX3CL1 and TNF-*α* data. However, as a control measurement, CX3CR1 immunostaining was performed on tissue specimens collected from each perfused placental lobule at the end of the adaptive period (30 min) and utilized as an initial (init.) measurement of CX3CR1 expression in the high-glucose and normal-glucose environments (groups HG and NG, resp.).

The standard immunohistochemical procedures were applied to visualize CX3CR1. Rabbit polyclonal antibody IgG to CX3CR1 (ab8020; Abcam Inc., USA; 10 *μ*g/mL) was used as the primary antibody, and goat anti-rabbit IgG was used as the biotinylated secondary antibody (ab64256; Abcam; 0.5% *v/v*). The primary antireceptor antibodies were visualized using a StreptAB-Complex/HRP Duet Kit (Dako Cytomation, Glostrup, Denmark) following the procedure recommended by the manufacturer, with 3,3′-diaminobenzidine as the chromogen. In the negative controls, the polyclonal primary antibody was replaced with normal rabbit preimmune IgG diluted in PBS containing 3% bovine serum albumin at the same protein concentration.

### 2.6. Mean Density of Placental Microvessel Network

In view of the fact that CX3CR1 is also expressed in vascular endothelial cells, it was presumed that the accuracy of CX3CR1 expression measurement might be significantly influenced by the local differences in density of placental microvessels [35–37]. To avoid this discrepancy in the results, the identification of the vasculature elements in placental sections was performed using endothelial cell maker, rabbit polyclonal antibody anti-CD31 (dilution 1 : 50, Abcam Inc., Cambridge, MA, USA; ab28364). Next, a biotinylated goat anti-rabbit antibody was used as the secondary (Abcam Inc.).

The technique of the mean density of placental vessels assessment was described in detail elsewhere [38, 39]. Briefly, using light microscopy with computed morphometry for quantitative analysis (Quantimet 500C+ image analysis workstation provided by Leica, UK), the vascular/extravascular tissular index (V/EVTI) was estimated in calibrated areas of the placental sections. Each specimen (paraffin section) underwent three area analyses repeated by two experienced, independent observers. The single area measured with the picture analyzer amounted to 685199 *μ*m^2^ and the total number of specimens 36 per group. The picture analysis procedure consisted in a measurement of the total vascular area. Consequently, the total lumen area of all types of identified vessels was summed up in both groups. With the purpose of a minimizing disruption caused by technical errors, especially unaxial section of the vessel, the lowest value of Ferret's diameter was accepted as the diameter of single lumen. Thus, V/EVTI represents the ratio, which reflects the intensity of vascularization and is most closely correlated with the mean density of placental microvessels.

### 2.7. CX3CR1 Expression: Quantitative Immunohistochemical Analysis

CX3CR1-immunostained paraffin sections of the placental lobule specimens were subjected to quantitative immunohistochemistry using morphometric software (Quantimet 500C+, Leica, UK). Light microscopy was applied to capture the images. All morphometric procedures were carried out twice by two independent researchers, and the average values were recorded. The intensity of immunostaining was evaluated through two parameters: the mean color saturation and thresholding in grey level histograms. Thus, the expression of CX3CR1 corresponded to the total immunostained calibrated area of the sections examined, where color saturation was treated as the segmentation criteria for objects. The total analysed area of the single image was 138246 *μ*m^2^ at 200x magnification. In each group, 108 visual fields were analysed (three visual fields per each isolated lobule). To achieve the maximum accuracy of the measurements, stability of power supply during morphometric procedures was assured by the power supply unit (Seasonic Platinum-1050, USA) and controlled with professional LCR (L—inductance, C—capacitance, and R—resistance) digital multimeter (CEM 9935, China) using back-probing power connectors of the computer's motherboard. The process of warming up and operating temperature (allowed range: 40–55°C) of the central processing unit (CPU) were monitored with a dedicated software (Core Temp 1.0 RC6 freeware, Arthur Lieberman). Other parameters, including illumination, shading correction, hue, averaging of image intake, luminescence, and the relation of illumination to quantification of area percentage of positively staining structures were standardized and monitored at the level of the morphometric software as described previously [33, 40]. Finally, the results obtained for the specimens examined were adjusted for the mean density of the placental microvessel network. The morphometric results that included the 90% confidence intervals were reported as the mean percentage values ± SEM.

### 2.8. CX3CR1 Expression: CX3CR1 Protein Content in the Perfused Placental Tissue Homogenates

For the comparative assessment of placental CX3CR1 expression independent of endothelial cells and the mean vascular density, CX3CR1 protein levels were quantified in the placental (the perfused lobule) tissue homogenates following the termination of the perfusion experiments. The Human CX3CR1 ELISA Kit (sandwich ELISA; LifeSpan BioSciences Inc., Seattle, WA, USA; catalogue number LS-F9187) was used, with a detection range of 0.156–10 ng/mL and a sensitivity of <0.055 ng/mL, as declared by the manufacturer.

Before obtaining a lysate, the tissue was cut into approximately 1 mm^3^ cubes using a razor blade on a glass plate on ice. To gently disrupt the cells, the cubes were transferred into a handheld Potter S homogenizer (Sartorius Stedim Biotech GmbH). Ice-cold phosphoprotein lysis buffer containing 4 mM sodium pyrophosphate, 50 mM HEPES, 100 mM NaCl, 10 mM EDTA, 10 mM NaF, 2 mM NaVO_4_, 1 mM PMSF, 10% Triton X-100, 5 *μ*g/mL leupeptin, and 5 *μ*g/mL aprotinin was used. The liquefied tissue was poured into 1.5-mL tubes, and the tissue and cell debris were removed by centrifugation at 10,000 × g for 3 min at 4°C.

Placental tissue lysates were obtained as described for the assessment of NF-*κ*Bp65 protein levels (Section 2.4). Then, the cleared supernatant was subjected to in vitro quantitative measurements of CX3CR1 protein using 96-well plates and a microplate reader.

### 2.9. TNFR1/TNFRSF1A Protein Content in the Perfused Placental Tissue Homogenates

For the comparative assessment of TNF-*α* receptor type 1 (TNFR1/TNFRSF1A and CD120a) expression in perfused lobule tissue, placental homogenates were prepared after the termination of the perfusion experiments. The liquefied tissue was obtained in the same manner as described above for the CX3CR1 ELISA Kit. Then, the homogenates were centrifuged for 5 minutes at 5000 × g, and the cleared supernatant was subjected to in vitro quantitative measurements of TNFRSF1A protein using 96-well plates and a microplate reader. The Human ELISA Kit for tumor necrosis factor receptor superfamily, member 1A (TNFRSF1A; sandwich ELISA; Cloud-Clone Corp., Wuhan, People's Republic of China catalogue number SEB499Hu) was used, with a detection range of 15.6–1000 pg/mL and a sensitivity of <6.5 pg/mL, as declared by the manufacturer.

### 2.10. Statistical Analysis

Mann–Whitney *U* test was applied. The results are expressed as the mean ± SEM, medians, or mean percentage values ± SEM. The differences between the two groups (high-glucose versus normoglycemic conditions) were deemed statistically significant if *p* < 0.05.

## 3. Results and Discussion

There is a growing body of evidence indicating that the biological properties of resveratrol may be translated into beneficial pharmacologic activities in cardiovascular diseases, including these coexisting hyperglycemic states [30, 41, 42]. Thus, the ability of resveratrol to counteract the increasing deterioration of endothelial cell function related to local CX3CL1 overproduction and/or altered CX3CR1 expression may be crucial [42, 43]. The CX3CL1 levels are shown in Table 3 (see also the experimental setup in Figure 2).

There were no significant differences in the initial concentration of CX3CL1 (_init_CX3CL1) between the placentae perfused with hyperglycemic perfusion fluid and those perfused with normoglycemic perfusion fluid. The mean _init_CX3CL1 was 93.8 ± 24.2 pg/mL in the HG group and 96.3 ± 23.0 pg/mL in the NG group. The median, rounded to the nearest whole number, was 84 [95% confidence interval (CI) 55–125] pg/mL in the HG group and 82 (95% CI 54–120) pg/mL in the NG group (Table 3). During the 120 min observation period including four perfusion fluid specimen collections, the mean CX3CL1 concentration significantly increased in both groups. The addition of LPS to the perfusion fluid evoked a significantly (*p* < 0.05) stronger response in the HG group, and the mean CX3CL1 levels were significantly higher in the HG group than in the NG group at every measurement. Based on the comparisons of the mean CX3CL1 levels and the dynamics of the increase in CX3CL1 levels at consecutive time points between subgroups HG-A and NG-A and the respective controls [hyperglycemic and normoglycemic perfused without resveratrol (HG-RF and NG-RF, resp.; see Table 3)], we concluded that 10 *μ*M resveratrol does not affect CX3CL1 production. Although CX3CL1 levels were consistently higher in the HG group compared to the NG group, higher doses of resveratrol (50 and 100 *μ*M) lowered CX3CL1 production in a dose-dependent manner in both groups (see subgroups HG-B, HG-C, NG-B, and NG-C in Table 3). This effect was more evident in perfusion fluid specimens obtained from the normoglycemic group, where the percent reductions in CX3CL1 levels after the administration of the highest dose of resveratrol (subgroup NG-C) amounted to 22.10, 54.79, and 60.04% (at 90, 120, and 150 min, resp.), versus 10.11, 32.65, and 37.63% in the hyperglycemic subgroup C. These data also show that the activity of resveratrol accrues over time within the period of 60 min. The results of the present comparative study focused on CX3CL1/CX3CR1 signaling in high-glucose versus normal-glucose environments may be useful for better understanding these complex relationships in the context of the potential therapeutic applications of resveratrol. To the best of our knowledge, this is the first publication on these issues pertaining to humans. The significantly higher levels of CX3CL1 in the HG group after LPS administration suggest that the hyperglycemic environment sensitizes perfused placental tissues to proinflammatory stimuli; the initial production of CX3CL1 was comparable in the two groups. Even after taking into account that LPS administration elicited higher CX3CL1 levels in the HG group compared to the NG group, the effectiveness of resveratrol (at 50 and 100 *μ*M) in decreasing CX3CL1 production (the percent reduction) by perfused placental lobules was significantly weakened in hyperglycemic conditions. It is worth mentioning that both hyperglycemia and inflammation influence proteolytic shedding on endothelial cells [44, 45]. Thus, further studies are needed to investigate whether observed changes in CX3CL1 levels are associated predominantly with the modulation of mRNA synthesis or proteolytic shedding.

The TNF-*α* levels in the perfusion fluid samples are summarized in Table 4.

Striking parallels were observed in the profiles of TNF-*α* and CX3CL1 production. Similarly, LPS administration evoked a significantly greater (*p* < 0.05) increase in TNF-*α* production in the HG group compared to the NG group. At the next time points (90, 120, and 150 min), the levels of TNF-*α* in subgroups HG-A, HG-B, and HG-C remained higher (*p* < 0.05) compared to those in subgroups NG-A, NG-B, and NG-C, respectively. Higher doses of resveratrol (50 and 100 *μ*M) inhibited the production of TNF-*α* in a dose-dependent manner, whereas 10 *μ*M resveratrol did not show this inhibitory effect. TNF-*α* levels and the dynamics of the increase in TNF-*α* levels at consecutive time points were not significantly different (*p* > 0.05) between subgroups HG-A and NG-A and the respective resveratrol-free controls: hyperglycemic (HG-RF) and normoglycemic (NG-RF) (see Table 4).

In contrast to CX3CL1, the time-dependent increase (within 60 min) in the inhibitory effect of 50 and 100 *μ*M resveratrol on TNF-*α* production in the perfused placental lobule was similar in the hyperglycemic and normoglycemic environments. The percent reductions in TNF-*α* levels for the highest dose of resveratrol were 25.85, 49.43, and 55.69% in subgroup NG-C and 34.67, 44.95, and 46.35% in subgroup HG-C (at 90, 120, and 150 min, resp.).

Because TNF-*α* is a strong inducer of CX3CL1 and influences CX3CR1 expression, the decelerated decrease in CX3CL1 levels under hyperglycemic conditions may reflect the modulation of CX3CR1 [22]. It is likely that the resveratrol-dependent reduction in TNF-*α* synthesis directly or indirectly triggers an autoregulatory mechanism that influences CX3CR1 expression in endothelial cells in response to the local decrease in CX3CL1 levels [22, 23].

**Figure 3:**
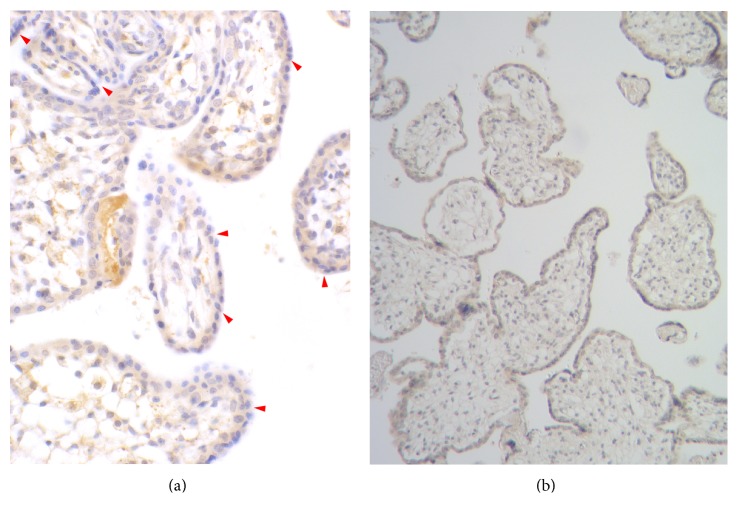
Immunohistochemical visualization of the receptor CX3CR1 in perfused placental tissue at 200x magnification (a) with the negative control ((b); mag. 100x). The image captured through optical microscope was digitally transformed for morphometric purposes. Immunostain-positive focal regions to a large extend correspond to the vascular endothelium (*arrowheads*). A single visual field observed with the naked eye looks substantially uniformly within each studied group. For that reason, only one immunostaining is presented for CX3CR1.

**Figure 4:**
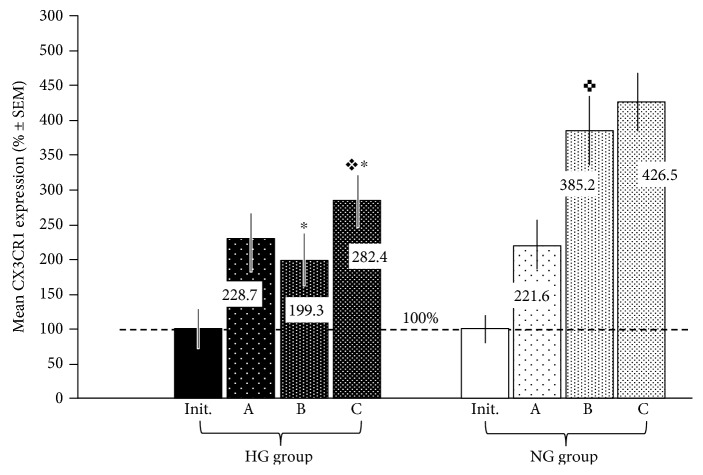
Effect of resveratrol administration on CX3CR1 expression. Resveratrol was given in three different doses (10, 50, and 100 *μ*M) in the respective subgroups (A, B, and C), and the mean placental expressions of CX3CR1 in perfused human placental tissue under hyperglycemic (glucose 25 mmol/L; HG group) and normoglycemic (glucose 5 mmol/L; NG group) conditions have been analysed. The mean value of CX3CR1 expression in the specimens obtained at the end of the adaptive period (before the administration of LPS; see Figure 2.) was treated as the initial (init.) and taken as 100% in each group. ∗ indicates *p* < 0.05 (HG group versus NG group); ^✜^ indicates *p* < 0.05 (HG subgroup C versus NG subgroup B); ^✚^ indicates *p* < 0.05 (NG subgroup B versus NG subgroup A).

The overall quality of CX3CR1 immunostaining in placental sections was sufficient for the application of standardized quantitative immunohistochemistry to investigate the expression of this receptor (Figure 3). Regions of the visual field demonstrating the strongest immunostain-positive reaction, captured from calibrated areas of the placental sections for computer image analysis, were to a large extent localized within the microvascular endothelium. This may confirm the appropriateness of analysing CX3CR1 expression in combination with V/EVTI. The differences in the initial expression of CX3CR1 were not significant between the groups, whereas resveratrol administration into the placental lobule vascular system after pretreatment with LPS significantly affected CX3CR1 expression in both groups (Figure 4). A positive correlation between resveratrol dose and CX3CR1 upregulation was documented in the NG group. The mean percent expression of CX3CR1 was significantly (*p* < 0.05) higher in subgroups NG-B and NG-C compared to subgroups HG-B and HG-C (385.2 and 426.5% versus 199.3 and 282.4%, resp.). After treatment with the lowest dose of resveratrol (10 *μ*M), the mean percent expression of CX3CR1 was similar in hyperglycemic and normoglycemic placental sections (228.7% in IA versus 221.6% in IIA).

Considering that 10 *μ*M resveratrol had no effect on CX3CL1 production in this study, LPS alone may be responsible for the upregulation of CX3CR1 in subgroups HG-A and NG-A. It is worth noting that in hyperglycemic subgroup B, the mean percent expression of CX3CR1 was similar (or even lower) than that in HG-A, while CX3CR1 expression was significantly upregulated in the normoglycemic subgroup B, with no significant difference compared to NG-C.

**Figure 5:**
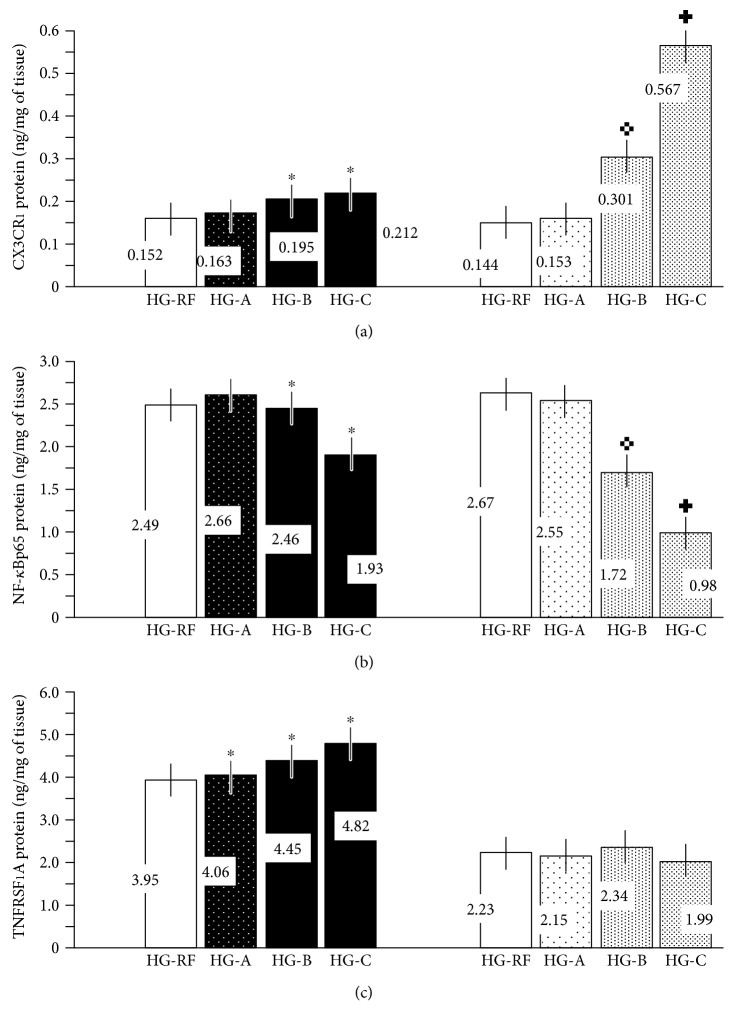
Contents of NF-*κ*Bp65, CX3CR1, and TNFRSF1A ((a), (b), and (c), resp.) in the lysates of the placental tissue. High-glucose (HG-A, HG-B, and HG-C) versus normoglycemic (NG-A, NG-B, and NG-C) subgroups. Resveratrol-free controls (HG-RF and NG-RF) are also included. The isolated placental lobules were perfused under hyperglycemic (glucose 25 mmol/L; HG group) and normoglycemic (glucose 5 mmol/L; NG group) conditions after the administration of resveratrol in three different doses (10, 50, and 100 *μ*M) in the respective subgroups (A, B, and C). ^∗^*p* < 0.05 (HG group versus NG group); ^✜^*p* < 0.05 (NG subgroup B versus NG subgroup A); ^✚^*p* < 0.05 (NG subgroup C versus NG subgroup B).

Thus, the increased mean CX3CR1 expression after resveratrol treatment may signify a dose-dependent compensatory mechanism. In all probability, the placental pool of endothelial cells dominates the other CX3CR1 compartments (other cell types) in placental tissue subjected to extracorporeal perfusion. The results obtained in the NG group (subgroups B and C) from quantitative CX3CR1 immunohistochemistry and CX3CR1 protein content measurements in the lysates (Figure 5(a)) indicate that a greater decrease in CX3CL1 production evokes higher (compensatory) expression of CX3CR1. Thus, the highest increase in mean CX3CR1 expression occurred in the normoglycemic subgroups. In the HG group, the discrepancy between the mean CX3CR1 expression in immunostained placental samples and the CX3CR1 protein content in the placental lysates may be explained by the fact that the mean microvascular density was considered and standardized using the V/EVTI parameter during the quantitative morphometry (see Section 2).

However, it is not clear whether the hyperglycemic environment itself upregulates CX3CR1/CX3CL1 signaling, as was reported in streptozotocin-induced diabetic rat kidneys [46].

The other placental lysates were used to determine NF-*κ*Bp65 protein levels as an indicator of the protein complex that controls cellular responses and cell survival by cytokine production (Figure 5(b)). Higher doses of resveratrol (50 and 100 *μ*M) significantly decreased NF-*κ*Bp65 protein content in placental tissue in the NG group in a dose-dependent manner, while the differences did not reach statistical significance in the HG group (*p* > 0.05). The placental tissue lysates from subgroups HG-A and HG-B contained the same amount of NF-*κ*Bp65 protein, further suggesting the lack of activity of 10 *μ*M resveratrol.

Hyperglycemia significantly influences a protein complex that controls DNA transcription, cytokine production, and cell survival and activates NF-*κ*B via an aldose reductase-controlled pathway [47]. Aldose reductase is considered an important factor in hyperglycemia-induced metabolic changes that lead to the development of secondary diabetic complications [48].

Interestingly, resveratrol reduces oxidative stress in diabetes through both deacetylation and inhibition of aldose reductase [43, 49]. The deacetylation of NF-*κ*Bp65 is a direct consequence of sirtuin-1 (SIRT-1) activation by resveratrol [43]. Thus, a high-glucose environment may be a special target for the therapeutic implementation of resveratrol. In our study, the dose-dependent response of NF-*κ*Bp65 content in perfused placental tissue lysates to higher doses of resveratrol (50 and 100 *μ*M) reached statistical significance (*p* < 0.05) only in the normoglycemic group. This difference between the groups may suggest that the antioxidant and anti-inflammatory potential of resveratrol is significantly reduced in a high-glucose environment. It is worth noting that NF-*κ*Bp65 content in the lysates of placental tissue perfused without resveratrol (HG-RF and NG-RF; see Figure 5(b)) was similar in both groups, and the mean values did not differ significantly from those reported for subgroups HG-A and NG-A. These data may seem unexpected because NF-*κ*B belongs to the category of “rapid-acting” primary transcription factors, that is, factors that are present in cells in an inactive state and do not require new protein synthesis to become activated and thus modify cellular responses to harmful stimuli [50]. However, we used normal placentae derived from nondiabetic mothers in this study. An increased protein content of NF-*κ*B family members has been reported in tissues, including the placenta, from diabetic patients [51, 52].

Finally, we observed a significant difference (*p* < 0.05) in TNFRSF1A protein levels in placental lysates from the two groups (Figure 5(c)). In contrast to CX3CR1 and CX3CL1 production, the decrease in TNF-*α* production in response to resveratrol (50 and 100 *μ*M) did not significantly affect TNFRSF1A protein content in the perfused placental tissue. The mean TNFRSF1A protein concentration was higher in the hyperglycemic environment (HG group) than in the normoglycemic environment (NG group). The differences between the respective subgroups (A, B, and C) within groups HG and NG were not significant, suggesting no effect of resveratrol on TNFRSF1A expression. In this situation, hyperglycemic conditions per se may be responsible for the consistently higher TNFRSF1A protein expression in all placental tissues obtained from group I [10, 53]. Alternatively, the content of this ubiquitous protein, one of the major receptors for TNF-*α*, in perfused placental tissue lysate may not reflect more subtle changes (e.g., in vascular endothelial cells), and perhaps another approach, such as a different research model, is needed in future studies. It is important to note that TNFRSF1A exists as both a cell surface receptor (the membrane-bound form; mTNFRSF1A) and a soluble protein (sTNFRSF1A) that competes with TNF-*α* for receptor sites on cells [27]. The replacement of circulating blood with perfusion fluid within the isolated placental lobule in our study could affect the interplay between the two forms of TNF-*α* receptor type I, thereby influencing the response to resveratrol. The involvement of sTNFRSF1A in the pathomechanism of hyperglycemia-induced renal disease was suggested recently in an advanced form of diabetic nephropathy [54].

Moreover, the content of other receptors within the TNF receptor superfamily that are expressed in endothelial cells, such as TNFR2, was not evaluated in this study. TNFR2 exists in a membrane-bound form (mTNFR2) and a soluble form (sTNFR2) and deserves attention because the analyses of TNFRSF1A knockout mice clearly indicated that TNFR2 stimulation alone is sufficient to activate most of the signaling pathways mediated via TNFRSF1A [27].

It is important to highlight that our results are strongly influenced by the experimental model. First and foremost, the circulating blood in the vascular system of the isolated lobule was replaced with perfusion fluid. In this system, the lack of blood, which provides a rich source of cytokines, enabled a more selective and simple interpretation of the observed relationships between CX3CL1/CX3CR1 and TNF-*α* and TNFRSF1A and NF-*κ*Bp65, both at the endothelial cell level and within placental tissue. However, the elimination of cytokines, activated leukocytes, platelets, and insulin circulating in the blood may create an imperfect model that limits the likelihood of the direct implementation of these results in clinical studies [55]. For example, hyperinsulinemia induced by a high-glucose environment enhances the transcriptional activity of NF-*κ*B, while in animal studies, resveratrol has been shown to improve insulin sensitivity, glucose tolerance, and lipid profiles in obese and/or metabolically abnormal animals [42, 56]. Finally, we used hyperglycemic perfusion fluid (HG group) to perfuse placental lobules isolated from women who experienced a normal, nondiabetic pregnancy. Obviously, as a complex metabolic abnormality, diabetes differs from hyperglycemic conditions generated experimentally and studied extracorporeally in a perfused placental lobule. It is likely that gestational diabetes mellitus (GDM), which develops relatively late during pregnancy, best fits our model of a hyperglycemic state. Pathologic changes in the microvasculature of a diabetic placenta, including abnormal angiogenesis and increased vascular density in the more advanced stages of diabetes, may influence cytokine levels and resveratrol activity [57].

In conclusion, because serum levels of CX3CL1 are associated with markers of insulin resistance in gestational diabetes, we investigated the possibility that treatment with resveratrol would decrease the production of both CX3CL1 and TNF-*α*, a strong CX3CL1/CX3CR1 inducer, in placental circulation [2, 8, 22, 30, 31]. Interestingly, in light of our results, the reported therapeutic effects of resveratrol on reducing hyperglycemia in adults who are obese or who present with type 2 diabetes or impaired glucose tolerance may stem from reductions in CX3CL1 and TNF-*α* production [28, 58]. Thus, the anti-inflammatory effects of resveratrol pertaining to CX3CL1/CX3CR1 signaling in placental circulation are expected to be optimal in pregnant individuals presenting with metabolic disorders under euglycemic conditions. Therefore, adequate control of glucose levels throughout diabetic pregnancy is crucial for obtaining the expected therapeutic effects of resveratrol. Supplementary treatment with resveratrol in diabetic pregnancy has promise to improve endothelial function, placental blood flow, and metabolism. Future studies are needed on diabetic placentae and diabetic pregnant subjects, including those that address safety issues and side effects, especially the potentially harmful actions of resveratrol on fetal pancreatic cells that may lead to their proliferation [59]. The hormetic nature of resveratrol in different types of human cell lines, which manifests as a biphasic dose response with stimulation or beneficial effects at low doses and inhibitory or toxic effects at high doses, must be considered when determining the optimal dose of this compound [60].

## Figures and Tables

**Table 1:** Clinical characteristics of the two studied groups.

Parameter	HG group (high glucose), NG group (normal glucose)
Number of patients/newborns/placentae/isolated lobules (*N*)	36/36/36/36 per group
Age of the patients in full years (range; mean; median)	24–32, 27, 28
Parity	0
Gestational age in days (range; mean; median)	269–285, 277, 278
Method of delivery	Cesarean section
Blood pressure during pregnancy	All records within normal range^a^
Proteinuria during pregnancy	Not present
Liver blood tests (aminotransferases, enzymes AST and ALT levels)	Within normal range^b^
Smoking during pregnancy	None declared active smoking
Diabetes during pregnancy	Not present
Body mass index < 21 or >35	None
Mother's blood (III trimester): hematocrit (Ht), hemoglobin (Hb), red blood cell (RBC) count, mean corpuscular hemoglobin concentration (MCHC)	All within normal ranges^c^
Other identified risk factors	None
Birth weight in grams (range; mean; median)	2960–3790, 3310, 3350
Sex of newborns (M—male, F—female)	19M + 17F
Weight of placenta in grams (range, mean, median)	545–774, 655, 663
Weight of isolated lobule in grams (range, mean, median)	HG group: 84–113, 103, 100; NG group: 87–111, 102, 103

Considering that each placenta was used for the isolation of two similar lobules for HG and NG groups, the groups may be treated as homogenous. ^a^The normal range of blood pressure was defined as systolic pressure between 100 and 140 mmHg and diastolic pressure between 60 and 90 mmHg. ^b^The normal range of values for AST is 5–40 units per liter of serum and, the normal range of values for ALT is 7–56 units per liter of serum. ^c^Hb levels 10.0–13.5 g/dL, RBC count 3.2–4.4 × 10^−6^/*μ*L, MCHC 319–355 g/L, and Ht 31–41%.

**Table 2:** Criteria for monitoring of correctness of extracorporeal fetal-side placental perfusion.

(1) Perfusion pressure: after the adaptative phase, pressure should be maintained within 7–11 kPa. Avoidance of rapid changes in blood pressure. Pressure < 7 kPa may be a result of vascular wall rupture. Pressure > 11 kPa suggests occlusion (clot and embolus)
(2) Flow stability: flow velocity 15–20 mL/min may change by no more than ±10% within 30 s
(3) Perfusion fluid volume: perfusion fluid loss after adaptive phase max. 5 mL/h
(4) pH–log [H^+^]: after 30, 90, and 150 min of perfusion, pH should not be <7.35, 7.30, and 7.25 in the arterial part of the system and at least 7.20, 7.15, and 7.10 in the venous part, respectively

**Table 3:** CX3CL1 levels (pg/mL) in the perfusion fluid samples collected at the consecutive time points.

Time point	Group							
	(Glucose 25 mmol/L)				(Glucose 5 mmol/L)			
	HG-RF		HG		NG-RF		NG	

Adaptive phase								
30 min (init.)								
Mean	89.5 ± 25.1		93.8 ± 24.2		91.6 ± 21.9		96.3 ± 23.0	
Median	86		84		83		82	
95% CI	57–129		55–125		51–126		54–120	
Experimental phase: after the administration of LPS (10 ng/mL)								
60 min								
Mean	380.4 ± 34.7		389.8 ± 37.9^★^		329.5 ± 33.2		335.4 ± 36.5	
Median	397		392		343		344	
95% CI	289–443		301–459		261–405		257–398	
Time point	Group/subgroup							
	HG-RF	HG-A	HG-B	HG-C	NG-RF	NG-A	NG-B	NG-C

Experimental phase: after the administration of resveratrol in three concentrations 10, 50, and 100 *μ*M (the subgroups A, B, and C, resp.)								
90 min								
Mean	449.8 ± 43.6	463.1 ± 49.1^★✜^	417.0 ± 32.9^★^	404.3 ± 45.6^★^	385.6 ± 39.4	381.2 ± 41.7^✜^	359.4 ± 36.3	300.4 ± 34.2
Median	455	458	414	404	375	374	352	296
95% CI	384–517	379–533	349–480	326–478	317–439	322–436	303–407	248–339
	% change from HG-RF	+2.95	−7.29	−10.11	% change from NG-RF	−1.14	−6.79	−22.10
120 min								
Mean	533.3 ± 54.2	538.3 ± 53^★✚^	454.3 ± 45.2^★❖^	359.2 ± 38.8^★^	425.6 ± 42.1	420.7 ± 39.1^✜✚^	268.4 ± 29.3^❖^	192.4 ± 20.2
Median	552	549	450	363	427	423	264	192
95% CI	442–637	457–629	357–526	314–409	349–470	358–474	202–311	143–245
	% change from HG-RF	+0.94	−14.81	−32.65	% change from NG-RF	−1.15	−36.94	−54.79
150 min								
Mean	530.2 ± 51.8	521.7 ± 55.4^★✚^	404.6 ± 47^★❖^	330.7 ± 36.6^★^	418.7 ± 38.5	419.1 ± 44.9^✜✚^	221.5 ± 22.4^❖^	167.3 ± 19.5
Median	507	511	399	332	419	414	225	169
95% CI	430–598	425–602	330–473	285–379	344–469	332–487	182–257	123–211
	% change from HG-RF	−1.60	−23.69	−37.63	% change from NG-RF	+0.1	−47.1	−60.04

Mean ± SEM, median (rounded to the nearest whole number), and 95% confidence interval (95% CI) are shown; HG-RF: high glucose resveratrol-free (control); NG-RF: normal glucose resveratrol-free (control); init.: initial concentration; ^★^*p* < 0.05 (high-glucose [HG] group versus normal-glucose [NG] group, including subgroups HG-A versus NG-A, HG-B versus NG-B, and HG-C versus NG-C, when applicable); ^✜^*p* < 0.05 (the subgroups A versus C within HG or NG group); ^✚^*p* < 0.05 (the subgroups A versus C and A versus B within HG or NG group); ^❖^*p* < 0.05 (the subgroups B versus C within HG or NG group).

**Table 4:** TNF-*α* levels (pg/mL) in the perfusion fluid samples collected at the consecutive time points.

Time point	Group							
	(Glucose 25 mmol/L)				(Glucose 5 mmol/L)			
	HG-RF		HG		NG-RF		NG	

Adaptive phase								
30 min (init.)								
Mean	46.3 ± 24.8		49.5 ± 25.5		40.2 ± 21.1		37.3 ± 18.7	
Median	44		46		40		39	
95% CI	32–99		34–102		27–78		26–81	
Experimental phase: after the administration of LPS (10 ng/mL)								
60 min								
Mean	158.4 ± 22.7		155.7 ± 20.1^★^		98.7 ± 16.6		98.7 ± 16.6	
Median	156		157		95		97	
95% CI	83–269		71–294		74–207		69–213	
Time point	Group/subgroup							
	HG-RF	HG-A	HG-B	HG-C	NG-RF	NG-A	NG-B	NG-C

Experimental phase: after the administration of resveratrol in three concentrations 10, 50, and 100 *μ*M (the subgroups A, B, and C, resp.)								
90 min								
Mean	269.7 ± 24.3	278.1 ± 24.4^★✜^	234.0 ± 18.3^★❖^	176.2 ± 16.9^★^	159.4 ± 17.7	166.3 ± 18.7^✜^	127.5 ± 19.4	118.2 ± 14.9
Median	272	274	237	173	169	170	124	115
95% CI	149–358	145–375	152–280	131–268	121–232	119–229	83–177	78–158
	% change from HG-RF	+3.11	−13.24	−34.67	% change from NG-RF	+4.33	−20.01	−25.85
120 min								
Mean	306.1 ± 30.2	301.9 ± 28.2^★✚^	228.9 ± 20.6^★❖^	168.5 ± 15.1^★^	201.3 ± 19.5	197.6 ± 17.2^✚^	133.2 ± 24.3	101.8 ± 11.2
Median	299	296	230	171	204	202	129	104
95% CI	159–400	165–404	169–288	121–242	133–264	135–257	73–164	57–146
	% change from HG-RF	−1.37	−25.22	−44.95	% change from NG-RF	−1.84	−33.83	−49.43
150 min								
Mean	287.6 ± 25.8	296.6 ± 24.3^★✚^	231.4 ± 20.5^★❖^	154.3 ± 13.7^★^	197.7 ± 18.9	201.8 ± 16.5^✚^	140.9 ± 13.7^❖^	87.6 ± 9.4
Median	307	304	227	152	197	196	141	92
95% CI	154–402	149–407	167–296	119–223	110–303	100–305	94–199	35–156
	% change from HG-RF	+3.13	−19.54	−46.35	% change from NG-RF	+2.07	−28.73	−55.69

Mean ± SEM, median (rounded to the nearest whole number), and 95% confidence interval (95% CI) are shown; HG-RF: high glucose resveratrol-free (control); NG-RF: normal glucose resveratrol-free (control); init.: initial concentration; ^★^*p* < 0.05 (high-glucose [HG] group versus normal-glucose [NG] group, including subgroups HG-A versus NG-A, HG-B versus NG-B, and HG-C versus NG-C, when applicable); ^✜^*p* < 0.05 (the subgroups A versus C within HG or NG group); ^✚^*p* < 0.05 (the subgroups A versus C and A versus B within HG or NG group); ^❖^*p* < 0.05 (the subgroups B versus C within HG or NG group).

## Abbreviations

ADAMs: Desintegrin and metalloproteinases
ADAM-10: Disintegrin and metalloproteinase (ADAM) 10
ADAM-17: Desintegrin and metalloproteinase (ADAM) 17
Act: Serine/threonine kinase Akt (protein kinase B, PKB)
ALT: Alanine aminotransferase
AST: Aspartate aminotransferase
BDNF: Brain-derived neurotrophic factor
BLC: Chemokine CXCL13
CPU: Central processing unit
CX3CL1: Chemokine CX3CL1 (fractalkine and neurotactin)
CX3CR1: Chemokine CX3CL1 receptor 1
DMSO: Dimethyl sulfoxide
ELISA: Enzyme-linked immunosorbent assay
ENA-78: Chemokine CXCL5
FGF-4: Fibroblast growth factor 4
G-CSF: Granulocyte colony-stimulating factor
GDM: Gestational diabetes mellitus
GM-CSF: Granulocyte-macrophage colony-stimulating factor
HG: High glucose
IFN-*γ:*: Interferon gamma
IKK: I*κ*B kinase complex
IL: Interleukin
_init_CX3CL1: Initial concentrations of CX3CL1
_init_TNF-*α*: Initial concentrations of TNF-*α*
IP-10: Chemokine CXCL10 (interferon gamma-induced protein 10; small inducible cytokine B10)
JAK/STAT: Janus kinase/signal transducers and activators of transcription
LPS: Lipopolysaccharide
MCP-1-3: Monocyte chemotactic proteins
MDC: Macrophage-derived chemokine
MIP-1*α*, MIP-1*β*, MIP-1*δ*: Macrophage inflammatory proteins 1: alpha, beta, delta
NF-*κ*B: Nuclear factor kappa-light-chain-enhancer of activated B cells
NG: Normal glucose
NIK: NF-*κ*B-inducing kinase
NK: Natural killer cells
PARC: Pulmonary and activation-regulated chemokine (chemokine CCL18)
PDGF: Platelet-derived growth factor
PDK1: Pyruvate dehydrogenase kinase 1 (pyruvate dehydrogenase [acetyl-transferring] kinase isozyme 1)
PBS: Phosphate-buffered saline
PI3-kinase: Phosphoinositide 3-kinase (phosphatidylinositol-4,5-bisphosphate 3-kinase)
*p*O_2_: Oxygen partial pressure
RANTES: Regulated on activation, normal T-cell expressed and secreted (chemokine CCL5)
SCF: Stem cell factor
TARC: Thymus- and activation-regulated chemokine (chemokine CCL17)
TGF-*β*: Transforming growth factor *β*
TIMP-1-2: Tissue inhibitors of metalloproteinases
TNFRSF1A: Tumor necrosis factor receptor superfamily member 1A (tumor necrosis factor receptor 1; CD120a)
TNF-*α*, TNF-*β*: Tumor necrosis factors: alpha, beta
TPO: Thyroid peroxidase
VEGF: Vascular endothelial growth factor
V/EVTI: Vascular/extravascular tissular index.
